# Serum Lipoprotein Profiles and Response to Pegylated Interferon Plus Ribavirin Combination Therapy in Patients With Chronic HCV Genotype 1b Infection

**DOI:** 10.5812/hepatmon.8988

**Published:** 2013-05-27

**Authors:** Yoshio Aizawa, Noritomo Shimada, Hiroshi Abe, Nobuyoshi Seki, Yuta Aida, Haruya Ishiguro, Makiko Ika, Keizo Kato, Akihito Tsubota

**Affiliations:** 1 Department of Gastroenterology and Hepatology, Jikei University School of Medicine, Tokyo, Japan; 2 Department of Gastroenterology and Hepatology, Shinmatsudo Chuo General Hospital, Chiba, Japan; 3 Institute of Clinical Medicine and Research, Jikei University Kashiwa Hospital, Chiba, Japan

**Keywords:** Hepatitis C, Ribavirin, Lipoproteins, Lipoproteins VLDL, Chromatography, High Pressure Liquid

## Abstract

**Background:**

Abnormal serum lipid profiles have been noted in patients with chronic hepatitis C virus (HCV) infection. Moreover, many reports suggest that serum lipoprotein profiles are more profoundly distorted in patients with HCV G1b infection who have an unfavorable response to pegylated interferon (peg-IFN) plus ribavirin (RBV) combination therapy. However, after the discovery of single nucleotide polymorphisms near the IL28B gene (rs8099917 and rs12979860) as potent predictive factors affecting the response to peg-IFN plus RBV, lipid factors are thought to be confounding factors.

**Objectives:**

To re-examine the significance of lipoprotein profiles on virological response to peg-IFN plus RBV combination therapy in patients with chronic HCV G1b infection, we examined cholesterol and triglyceride concentrations in each lipoprotein fraction separated by high performance liquid chromatography.

**Patients and Methods:**

Lipoprotein profiles were examined using fasting sera from 108 patients infected with HCV G1b who had chronic hepatitis, as determined by liver biopsy. Results of lipoprotein profiles and clinical data, including IL28B genotype and amino acid substitution at aa70 of HCV G1b, were compared between patients with a sustained virological response (SVR) and non-SVR or a non-virological response (NVR) and virological responses other than NVR (non-NVR). In addition, significant predictive factors independently associated with virological response to peg-IFNα-2b plus RBV were determined by logistic regression analysis.

**Results:**

An increased ratio of cholesterol/triglyceride in very low-density lipoprotein (odds ratio (OR) 3.03; 95% confidence interval (CI) 1.01-9.44) along with a major genotype of rs8099917 (OR 9.09; 95% CI 2.94-33.33), were independent predictive factors for SVR. In contrast, lipid factors were not elucidated as independent predictive factors for NVR.

**Conclusions:**

Examination of the fasting lipid profile has clinical importance in predicting the efficacy of peg-IFN-α-2b plus RBV combination therapy for patients with HCV G1b even after the discovery of the IL28 genotype as a potent predictive factor.

## 1. Background

Hepatitis C virus (HCV) is a unique virus targeted towards liver cells; this virus closely interacts with host lipoprotein metabolism (1, 2). Very low-density lipoproteins (VLDL) synthesized and secreted from liver cells play a critical role in the generation and secretion of HCV (3). HCV particles are composed of HCV-lipoprotein complexes, referred to as lipoviral particles (LVP), in peripheral blood (4, 5). HCV particles in the plasma of patients with chronic HCV infection are thought to be associated with different classes of lipoproteins showing different buoyant density. The infectivity of HCV is stronger with low buoyant density LVP than with high buoyant density LVP, suggesting the significance of VLDL as a component of LVP on the HCV lifecycle (6). Lipoprotein profiles in the sera of patients with chronic HCV infection have been studied with special attention to the association of HCV genotype and the response to interferon (IFN)-based antiviral therapy (7-10). Abnormal lipoprotein profiles are more prominent in HCV genotype 3 (G3) than in HCV genotype 1 (G1) infection (11). Apolipoprotein B (Apo B)-related cholesterol is lower in patients with a non-favorable response to pegylated IFN (peg-IFN) plus ribavirin (RBV) therapy than in patients with a favorable response who are infected by HCV G1 (12). These findings suggest that investigation of lipoprotein profiles in patients with chronic HCV infection may be helpful in understanding the interaction of HCV and host lipoprotein metabolism. Lipoproteins in peripheral blood are usually examined using conventional simplified methods. High-density lipoprotein cholesterol (HDL-C) is usually determined by the precipitation method, whereas low-density lipoprotein cholesterol (LDL-C) is estimated indirectly by the Friedwald equation (13) or direct methods. However, it is difficult to measure VLDL cholesterol (VLDL-C) directly by routine laboratory tests. To determine serum lipid profiles more precisely, one of the most reliable methods is density gradient ultracentrifugation. However, ultracentrifugation requires a relatively large sample volume and a lot of time; thus it is not suitable for general use in clinical settings. Polyacrylamide gel electrophoresis or nuclear magnetic resonance is another method to assess lipid profiles (14, 15). In addition, high-performance liquid chromatography (HPLC) could be applied to differentiate lipoproteins based on the differences in particle diameter (16, 17).

## 2. Objectives

In the present study, we used a computer-assisted online dual detection method by HPLC that allows simultaneous determination of cholesterol and triglyceride (TG) profiles from a single injection of sample. The characteristics of lipoprotein profiles participating in the response to peg-IFNα-2b plus RBV combination therapy in patients with chronic HCV G1b infection were analyzed to re-examine the significance of lipoprotein profiles on the virological response to this treatment regimen.

## 3. Patients and Methods

### 3.1. Patients

Among patients with chronic HCV G1b infection diagnosed as chronic hepatitis by liver biopsy and treated with Peg-IFNα-2b plus RBV combination at Katsushika Medical Center and/or Kashiwa Hospital, the Jikei University School of Medicine, and Shinmatsudo Central General Hospital between April 2008 and November 2010, 120 Japanese patients were randomly asked to participate into this observational cohort study designed to evaluate the significance of host lipids on the response to anti-viral therapy, and 119 patients accepted our proposal. HCV genotype was confirmed using the conventional polymerase chain reaction (PCR)-based method. Patients with HBV co-infection, with cirrhosis or hepatocellular carcinoma, or co-infected with HIV were excluded. In addition, patients with diabetes mellitus or treated with a lipid-lowering drug were excluded. All enrolled patients were treated according to the treatment protocol based on response-guided therapy (18). Patients were treated with standard peg-IFN and ribavirin therapy according to the American Association for the Study of the Liver Diseases (AASLD) guidelines (19). Briefly, patients with chronic HCV G1b infection received subcutaneous peg-IFNα-2b (Peg-Intron^®^; MSD, Tokyo, Japan) at a dose of 1.5 µg/kg once weekly, and oral RBV (Rebetol^®^; MSD) at a dose of 600–1000 mg twice daily, adjusted according to body weight (600 mg for weight of 60 kg or less, 800 mg for weight of 60–80 kg or less, and 1000 mg for weight above 80 kg). The standard treatment duration lasted 48 weeks, although among patients with detectable HCV RNA at week 12, the treatment period was extended to 72 weeks. When serum HCV RNA levels were not decreased more than 2 logs at week 12 or HCV RNA was detectable at week 24 of therapy, treatment was discontinued prematurely and defined as a non-virological response (NVR). A virological response (VR) was defined as undetectable serum HCV RNA. Sustained virological response (SVR) was defined as undetectable serum HCV, 24 weeks post-treatment. Transient viral response (TVR) was defined as VR during treatment, but reappearance of serum HCV RNA during the follow-up period. Adherence to more than 80% of the scheduled doses during the first 12 weeks was required for inclusion in this study. Moreover, patients who discontinued treatment within 24 weeks of treatment for reasons other than virological failure were excluded. Finally, 108 out of the 119 patients were suitable for the inclusion criteria. Liver biopsy specimens were reviewed using the METAVIR scoring system for staging of fibrosis and grading of necro-inflammation activity (20). The serum HCV RNA was assessed using a quantitative PCR assay (COBAS TaqMan HCV test, Roche Diagnostics, Tokyo, Japan). Clinical and laboratory data were assessed at the time of the liver biopsy, before treatment, and fasting sera (taken early in the morning after at least 8 hours of fasting) were collected and used for detection of lipoprotein profiles. This study protocol was conducted in accordance with the provisions of the Declaration of Helsinki and Good Clinical Practice guidelines and was approved by the Institutional Review Board of each participating facility. Informed consent was obtained from all patients. Genotype of IL28B (rs8099917) was examined by our previously described method in 101 out of 108 HCV G1b patients (21). The major (TT) genotype of IL28B was noted in 69 patients and the minor (TG or GG) genotype was noted in 32 patients. Pre-treatment amino acid (aa) substitution at aa70 in the core region of HCV G1b, a viral factor affecting the therapeutic outcome, was analyzed by the method of Akuta et al. in 98 patients (8). Wild type (arginine, Arg70) was noted in 62 patients and non-wild type (glutamine/histidine, Gln70/His70) was noted in 36 patients. Both IL28B genotype and aa substitution at 70 were noted in 98 patients.

### 3.2. Analysis of Lipoprotein Profiles

**Figure 1.**
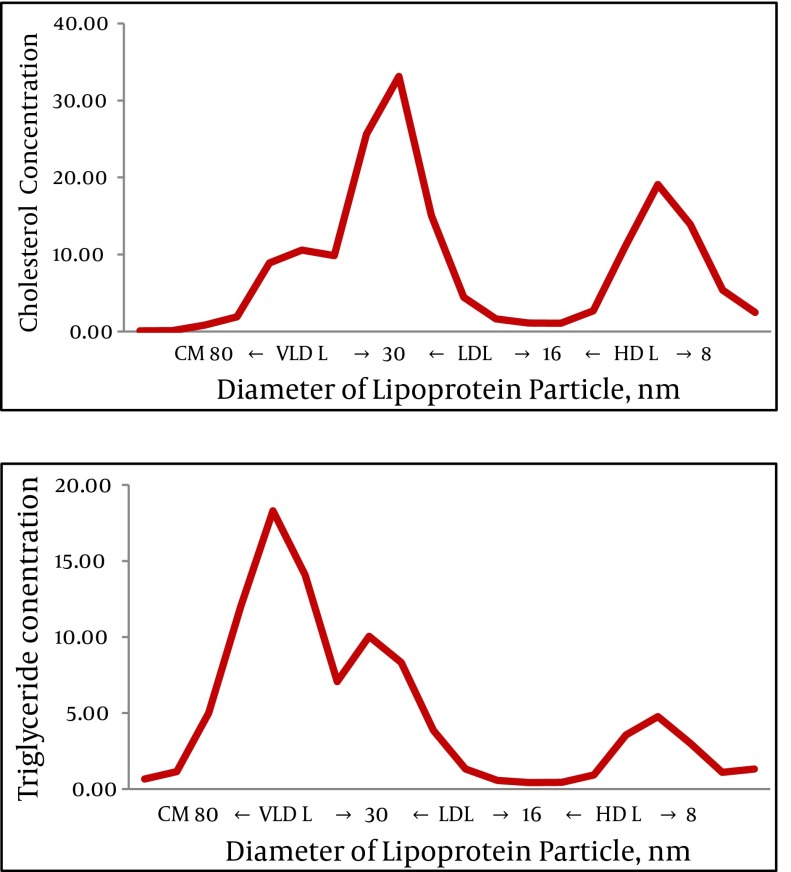
Pattern of Serum Lipoprotein Fractionated by HPLC with Online Enzymatic Dual Detection of Cholesterol and Triglyceride

Fasting serum lipoprotein profiles were analyzed by HPLC with online enzymatic dual detection of cholesterol and TG (Skylight Biotech, Inc., Akita, Japan) as described previously (16, 17). Briefly, a 10 µL whole serum sample was injected into two connected columns (300 × 7.8 mm) of TSKgel LipopropakXL (Tosoh, Tokyo, Japan) and eluted by TSKeluent Lp-1 (Tosoh). The effluent from the columns was divided by micro splitter and continuously monitored at 550 nm after an online enzymatic reaction with a commercial kit, Determiner L TC (Kyowa Medex Co. Limited, Tokyo, Japan) and Determiner L TG (Kyowa Medex Co. Limited). Then, the cholesterol and TG concentrations were calculated by a computer program. Lipoprotein particles fractionated into four major lipoproteins according to particle diameter were as follows: ≥80 nm was clarified as chylomicrons, from 30 nm ≤ to <80 nm as VLDL, from 16 nm ≤ to <30 nm as LDL, and from 8 nm ≤ to <16 nm as HDL. The concentrations of cholesterol and TG were measured in each major lipoprotein fraction and the cholesterol/TG (C/T) concentration ratio was calculated. The typical pattern of serum lipoprotein fractionated by this HPLC system is shown in Figure 1.

### 3.3. Statistical Analysis

Continuous variables are given as mean ± SD and categorical variables are given as numbers. Differences between two groups were evaluated using the Mann-Whitney U-test or the χ2 test with Yates’ correction. All *P* values less than 0.05 with a two-tailed test were considered significant. Variables that achieved statistical significance (*P* < 0.05) were entered into multivariate logistic regression analysis to identify independent predictive factors. The odds ratios (OR) and 95% confidence intervals (CI) were also calculated. STATISTICA version 8 (StatSoft Japan Inc., Tokyo, Japan) and Mintab 16 (Kozo Keikaku Engineering Inc., Tokyo, Japan) were used for statistical calculations. The *P* value was calculated to three decimal places and *P* < 0.001 was used for lower values.

## 4. Results

### 4.1. Lipoprotein Profiles and Clinical Data

Fasting serum lipoprotein profiles examined by online dual detection HPLC and clinical data of 108 patients are summarized in Table 1. The C/T ratio was low in TG rich lipoprotein (VLDL) fraction, and similar in LDL and HDL fractions.

**Table 1.** Clinical Features and Lipoprotein Profiles

Discrete Traits	n = 108
**Sex, No. (%)**	
Males	44 (41)
Females	66 (59)
**METAVIR Fibrosis Stage, No. (%)**	
1	56 (52)
2	34 (31)
3	18 (17)
**METAVIR Activity Grade, No. (%)**	
1	53 (49)
2	50 (46)
3	5 (5)
**Quantitative Traits**	
**Age, mean ± SD, y^a^**	58.1 ± 11.0
**HCV RNA, mean ± SD, log IU/mL**	6.4 ± 0.6
**ALT, mean ± SD, IU/L**	59.1 ± 42.5
**AST, mean ± SD, IU/L**	53.8 ± 37.6
**Albumin, mean ± SD, g/** **dL**	4.2 ± 0.3
**WBC, mean ± SD, X102/mm3**	52.3 ± 17.2
**Hb** **, mean ± SD, g/** **dL**	13.4 ± 1.8
**Platelet, mean ± SD, X104/mm3**	18.1 ± 6.0
**BMI, mean ± SD, kg/m3**	23.3 ± 2.1
**Lipids, mean ± SD**	
Total Cholesterol, mg/dL	164.3 ± 30.6
Triglycerides, mg/dL	90.6 ± 30.1
**VLDL, mean ± SD**	
Cholesterol, mg/dL	34.0 ± 10.6
Triglycerides, mg/dL	41.3 ± 18.0
C/T ratio	0.94 ± 0.47
**LDL, mean ± SD**	
Cholesterol, mg/dL	78.4 ± 19.6
Triglycerides, mg/dL	26.0 ± 7.8
C/T ratio	3.2 ± 0.9
**HDL, mean ± SD**	
Cholesterol, mg/dL	48.9 ± 14.1
Triglycerides, mg/dL	17.3 ± 6.2
**C/T ratio**	3.1 ± 1.1

^a^Abbreviations: ALT, alanine aminotransferase; AST, aspartate aminotransferase; BMI, body mass index; C/T ratio, cholesterol/triglyceride ratio; Hb, hemoglobin; HCV, hepatitis C virus; HDL, high-density lipoprotein; LDL, low-density lipoprotein; VLDL, very low-density lipoprotein; WBC, white blood cell; y, year

### 4.2. Responses to Peg-IFN-α-2b Plus RBV Therapy, Distribution of aa 70 Substitution, and IL28B Genotype

Fifty-six patients were classified as SVR, 22 were TVR, and 30 were NVR. The SVR rate was 51.9% and the NVR rate was 27.8%. In terms of aa substitution at 70 in the HCV G1b core region, among patients with SVR, 39 were Arg70 and 14 were Gln70/His70. In TVR patients, 14 were Arg70 and 5 were Gln70/His70, and in NVR patients, 9 were Arg70 and 17 were Gln70/His70. In terms of the IL28B (rs8099917) genotype defined as a single nucleotide polymorphism, among patients with NVR, only 4 were defined as the major genotype and 23 were the minor genotype. In patients with TVR, 16 were the major genotype and 4 were the minor genotype, and in patients with SVR, 49 were the major genotype and 5 were the minor genotype.

### 4.3. Factors Contributing to SVR in Chronic HCV G1b Patients

When pre-treatment clinical data (age, sex, alanine aminotransferase (ALT) level, albumin, quantity of HCV RNA, and platelet count), histological stage, activity score, substitution at aa70 in the core region of HCV, and IL28Bgenotype other than lipoprotein profiles, including those obtained by HPLC, were compared between patients with SVR and non-SVR (TVR plus NVR), a significant difference was found in distribution of the IL28B genotype, distribution of the aa70 substitution, serum ALT level, and the C/T ratio in VLDL (Table 2).

**Table 2.** Difference in Clinical Data Between Patients with Sustained Virological Response and Non-Sustained Virological Response

	SVR^a^, n = 56	Non-SVR, n = 52, No. (%)	*P*value
**Discrete Traits**			
**Sex, No. (%) **			0.067
Males	28 (50)	16 (31)	
Females	28 (50)	36 (69)	
**METAVIR Fibrosis Stage, No. (%) **			0.547
1	30 (53)	26 (50)	
2	15 (27)	19 (37)	
3	11 (20)	7 (13)	
**METAVIR Activity, No. (%)**			0.445
1	25 (45)	28 (54)	
2	28 (50)	22 (42)	
3	3 (5)	2 (4)	
**HCV Core aa70 (n = 98), No. (%)**			0.037
Arg70	39 (74)	23 (51)	
Gln70 or His70	14 (26)	22 (49)	
**IL28B Genotype (n = 101), No. (%)**			< 0.001
major	49 (91)	20 (43)	
minor	5 (9)	27 (57)	
**Quantitative Traits, mean ± SD**			
Age, y	58.0 ± 11.3	58.4 ± 10.9	0.795
HCV RNA, log IU/mL	6.38 ± 0.60	6.40 ± 0.46	0.594
ALT, IU/L	68.4 ± 51.4	49.3 ± 25.9	0.042
Albmin, g/dL	4.19 ± 0.31	4.14 ± 0.29	0.772
Platelet, X104/mm3	18.54 ± 5.83	17.66 ± 6.23	0.241
**Lipid, mean ± SD, mg/** **dL**			
Total Cholesterol	167.2 ± 28.1	161.2 ± 33.1	0.498
Triglycerides	87.8 ± 27.5	93.5 ± 32.6	0.658
**VLDL **			
Cholesterol, mean ± SD, mg/dL	35.7 ± 10.5	32.1 ± 10.6	0.056
Triglycerides, mean ± SD, mg/dL	42.5 ± 14.5	48.3 ± 21.0	0.241
C/T ratio	0.92 ± 0.40	0.75 ± 0.35	0.011
**LDL, mean ± SD**			
Cholesterol, mg/dL	80.1 ± 19.2	77.4 ± 20.2	0.448
Triglycerides, mg/dL	26.1 ± 7.1	25.9 ± 8.5	0.510
C/T ratio	3.15 ± 1.06	3.13 ± 0.86	0.894
**HDL, mean ± SD **			
Cholesterol, mg/dL	48.9 ± 11.8	49.7 ± 16.3	0.969
Triglycerides, mg/dL	16.9 ± 6.8	17.7 ± 5.6	0.242
**C/T ratio**	3.15 ± 1.06	2.99 ± 1.15	0.573

^a^Abbreviations: aa, amino acid; ALT, alanine aminotransferase; Arg, arginine; AST, aspartate aminotrasferase; C/T ratio, cholesterol/triglyceride ratio; Gln, glutamine; HCV, hepatitis C virus; His, histidine; HDL, high-density lipoprotein; IFN, interferon; LDL, low-density lipoprotein; SVR, sustained virological response; VLDL, very low-density lipoprotein

Multivariate logistic model was applied for the four variables that were significantly different between SVR and non-SVR to determine independent predictive factors. Among the 98 patients in whom both aa substitution of the HCV core 70 and IL28B genotype were examined, the IL28B major genotype (OR 9.09; 95% CI 2.94-33.33) and the C/T ratio of VLDL (OR 3.03; 95% CI 1.01-9.44) were elucidated as independent factors contributing to SVR. However, substitution at aa70 and serum ALT levels were not independent factors contributing to SVR (Table 3).

**Table 3.** Multivariate Logistic Regression Analysis of Factors Predicting Sustained Virological Response in Patients with Hepatitis C Virus G1b (n = 98)

Variable	Odds Ratio	95% CI^a^	*P*value
**ALT, IU/L**	1.02	1.00-1.03	0.058
**Arg70/Gln70 or** **His70**	2.27	0.79-6.67	0.129
**Major/Minor IL28B** **Genotypes**	9.09	2.94-33.33	< 0.001
**C/T Ratio in VLDL**	3.03	1.01-9.44	0.048

^a^Abbreviations: ALT, alanine aminotransferase; Arg, arginine; CI, confidence interval; C/T ratio, cholesterol/triglyceride ratio; Gln, glutamine; His, histidine; VLDL, very low-density lipoprotein

### 4.4. Factors Affecting NVR

When the study population, was divided into 30 patients with NVR and 78 patients with non-NVR (TVR plus SVR), a significant difference was found in the level of VLDL-C (28.8 ± 10.1 mg/dL vs 35.8 ± 10.3 mg/dL, *P* = 0.012), the C/T ratio of VLDL (0.82 ± 0.44 vs 0.99 ± 0.47; *P* = 0.045), distribution of IL28B genotype (major/minor, 4/23 vs. 65/9; *P* < 0.001), and substitution at aa70 (Arg70:Gln70/His70, 9:17 vs. 53:19; *P *= 0.001). However, the difference in LDL-C level was not significant (72.5 ± 21.4 mg/dL vs 80.5 ± 18.6 mg/dL; *P* = 0.158). After multivariate logistic analysis for extracting independent predictive variables in 98 patients who were determined to have both the genotype of IL28B and substitution at aa70, the minor IL28B genotype (OR 50; 95% CI 10-100) and Gln70/His70 in the core region of HCV (OR 6.25; 95% CI 1.52-25) were shown to be independent factors predicting NVR, but VLDL-C and the C/T ratio of VLDL were not (Table 4).

**Table 4.** Multivariate Logistic Regression Analysis of Factors Predicting Non-Virological Response in Patients with Hepatitis C Virus G1b (n = 98)

Variable	Odds Ratio	95% CI	*P*value
**Gln70 or His70/Arg70**	6.25	1.52-25	0.011
**Minor/Major** **IL28B Genotypes**	50	10-100	< 0.001
**VLDL Cholesterol,** **mg/dL**	0.97	0.88-1.06	0.487
**C/T Ratio in VLDL**	0.57	0.06-5.26	0.626

Abbreviations: CI, confidence interval; Arg, arginine; Gln, glutamine; His, histidine; VLDL, very low-density lipoprotein; C/T ratio, cholesterol/triglyceride ratio

## 5. Discussion

Disturbance of serum lipoprotein profiles in peripheral blood of patients with chronic HCV infection may play a critical role in cell entry of HCV and affect the efficacy of IFN-based antiviral therapy (22). In addition, the importance of lipid factors on treatment outcome of antiviral therapy may continue in the era of direct acting antivirals (DAA). DAA should be used in combination with IFN plus RBV because monotherapy with a newly developed DAA (protease inhibitor; boceprevir or telaprevir) causes rapid emergence of resistant HCV (23, 24). Therefore, factors affecting IFN plus RBV combination therapy such as lipid profiles may also influence the efficacy of triple therapy (DAA, IFN, and RBV). In the present study, we fractionated serum lipoprotein particles into chylromicrons, VLDL, LDL, and HDL using HPCL. This HPLC-based method required a small sample size and a relatively short time (about 25 minutes) for fractionation. Therefore, this HPLC-based method may be superior to other methods for detailed examination of lipoproteins in many patients. Furthermore, this HPLC system offers precise information on TG concentrations along with cholesterol levels in each fraction simultaneously (25). In a study on HCV G1, distortion of lipoprotein metabolism was more profound in patients who were non-favorable responders, and decreases of LDL-C, HDL-C, and TG were reported using conventional measurement technologies (26). However, after the discovery of rs8099917 and rs12979860 near the human IL28B gene as a strong host factor affecting the response to peg-IFN plus RBV therapy (27, 28), the significance of lipid profiles on predicting SVR was thought to be doubtful because the power of the IL28B genotype for predicting the efficacy of peg-IFN plus RBV therapy was so strong (29, 30). In the present study, we found that the C/T ratio in fasting serum VLDL was independently associated with SVR along with the IL28B genotype in patients with chronic HCV G1b who were treated with peg-IFNα-2b plus RBV. However, significant differences were not observed in other lipids including LDL-C. Using a similar method, Mawatari et al. showed that the cholesterol levels in the 44.5-nm and 36.8-nm subfraction of VLDL, in addition to the 25.5-nm subfraction of LDL, were significantly higher in patients with SVR than in non-SVR who were infected with HCV G1b and treated with peg-IFN plus RBV (31). However, they examined a relatively small number of patients without information on IL28B genotype. In our study, we did not subdivide the VLDL and/or LDL fraction because the VLDL and/or LDL fraction was not clearly separated into subfractions by the HPLC method as shown in Figure 1. It is known that there are at least two subtypes of VLDL: large, TG-rich VLDL1 and small VLDL2 (32). Therefore, increases of the C/T ratio in VLDL may indicate a decrease in VLDL1 and/or an increase in VLDL2. Based on the report of Mawatari et al., increases in cholesterol in the 44.5-nm and 36.8-nm subfractions of VLDL were associated with a favorable response to combination therapy (31). This finding may indicate an increase in the specific subtype of VLDL sized 36.8-44.5 nm. As shown in their report, TG levels of the 44.5-nm subfraction were concomitantly increased in patients with SVR. Because of the relative increase of cholesterol compared with TG, the C/T ratio of this subfraction was higher in patients with SVR than in patients with non-SVR. Thus, our finding of increased C/T ratio in VLDL does not conflict with the results of previous reports, and we assume that the increase of C/T ratio in VLDL fraction in patients with SVR was not merely due to increases or decreases in specific subtypes of VLDL but also to relative increases of cholesterol components in the VLDL particle having a similar diameter. Our finding that the difference of biochemical nature of VLDL may affect the response to peg-IFN plus RBV therapy is an independent phenomenon apart from the IL28B genotype. This issue has not been mentioned till now and we believe that our literature is the first report to refer to the significance of the biochemical nature of VLDL. The reason why the increase of C/T ratio in the VLDL fraction could be related to a favorable response to peg-IFN plus RBV is not fully understood. We assume that one of the reasons may be modulation of the immune function of dendritic cells by modified lipoproteins (33). In the present study, we also examined factors associated with NVR. However, no lipid factor was elucidated as an independent factor. In terms of predicting NVR, the IL28B genotype is an extraordinary strong factor, and aa substitution at 70 in the core region of HCV G1b is also a strong predictor. These factors may also affect serum lipid profiles, as described in our previous study (21). Therefore, lipid factors were not independent but thought to be confounding factors of the IL28B genotype and/or aa70 substitution for predicting NVR. In conclusion, the nature of VLDL expressed as C/T ratio is an independent factor in predicting a favorable response to peg-IFN plus RBV combination therapy in patients with chronic HCV genotype 1b infection.
